# Prof. Charcot

**Published:** 1879-08

**Authors:** 

**Affiliations:** Berlin, N. W. 6 Kronprinzen-Ufer den 29^ten^


### ARTICLE XI.

BERLIN, N. W. 6 Kronprinzen-Ufer den 29 ten May, 1879.

In the February number of the CHICAGO MEDICAL JOURNAL AND EXAMINER Dr. Caldwell gives an account of one of my lectures which he attended, and of a private conversation which he says I had with him about Prof. Charcot of Paris.

I hereby declare that I never authorized Dr. Caldwell to publish the substance of my lecture nor that of a private conversation. I further declare that the substance of my lecture is in part incorrectly reported, and that I cannot have used in speaking of Prof. Charcot, the expressions attributed to me by Dr. Caldwell, because they do not express my opinion.

Receiving frequent passing visits from foreign physicians I do not recollect the person of Dr. Caldwell nor the conversation to which he alludes, and therefore cannot say how this misunderstanding has arisen. I presume, unless I am to suppose an intentional misrepresentation on the part of Dr. Caldwell in his publication so contrary to all European notions of what is fitting, that the misunderstanding grew out of Dr. Caldwell's insufficient knowledge of German or out of my imperfect way of expressing myself in English. I do not know in what language the supposed conversation was conducted, and can only say that the English expressions which I am said to have used to the prejudice of Prof. Charcot were hitherto unknown to me.

Finally, I expressly declare that I have always felt and still feel the greatest respect and admiration for Prof. Charcot's scientific achievements and for his character as a man, and further that I never conceived the thought, much less expressed it, of passing such a judgment on his recent experiments in metalloscopy as Dr. Caldwell puts into my mouth.

DR. WESTPHAL.

